# ﻿Rapid and sensitive diagnosis of plant quarantine fungi *Alternariatriticina* and *Plenodomuslibanotidis* based on the RPA-CRISPR/Cas12a system

**DOI:** 10.3897/imafungus.16.153604

**Published:** 2025-06-10

**Authors:** Dorji Phurbu, Zhipeng Feng, Lei Cai, Fang Liu

**Affiliations:** 1 Xizang Key Laboratory of Plateau Fungi, Institute of Plateau Biology of Xizang Autonomous Region, Lhasa, China Institute of Plateau Biology of Xizang Autonomous Region Lhasa China; 2 State Key Laboratory of Microbial Diversity and Innovative Utilization, Institute of Microbiology, Chinese Academy of Sciences, Beijing, China Chinese Academy of Sciences Beijing China; 3 College of Life Sciences, University of Chinese Academy of Sciences, Beijing, China University of Chinese Academy of Sciences Beijing China

**Keywords:** Fluorescence detection, lateral flow detection, RPA, visual detection

## Abstract

With the increase in cross-border transmission in the context of globalization, the necessity for developing rapid and accurate detection methods for plant pathogens has become critical. This study introduces a recombinase polymerase amplification (RPA) technique combined with CRISPR/Cas12a cleavage and fluorescence-based detection systems (FRB) or paper-based lateral flow strips (PLFS) for the rapid on-site detection of invasive alien fungi, specifically *Alternariatriticina* and *Plenodomuslibanotidis*, which pose significant threats to agriculture and biodiversity. The results demonstrate that either RPA-CRISPR/Cas12a-FRB or RPA-CRISPR/Cas12a-PLFS can accurately detect the target species within 30 min, with a sensitivity of up to 10 pg/μL. These portable and easy-to-use assays are suitable for rapid on-site screening of plant pathogenic fungi in plant tissues, enabling applications in disease control and port quarantine.

## ﻿Introduction

The invasion of alien species is a major issue and a serious challenge faced globally, concerning food security, environmental disasters, and human health. According to a 2023 article published by the World Economic Forum, a four-year assessment conducted by 86 researchers from 49 countries estimates that the global economic losses caused by invasive species amount to approximately $432 billion annually (https://www.weforum.org/stories/2023/09/invasive-species-cost-global-economy-billions/). In particular, with the rapid development of economic globalization and regional integration, the frequency of cross-border transmission and spread of potentially imported alien pests has increased dramatically, and the increase in the number of alien species shows no sign of saturation (Seebens et al. 2017). Especially, the spread of invasive species across countries and continents is a major driver of biodiversity loss, disrupting the complex web of ecosystems, and contributing to 60% of recorded plant and animal extinctions. Therefore, preventing the arrival of pathogens into new regions is the best way to manage the threat of invasive species.

A key prerequisite for prevention of potential invasive species is to accurately identify them and implement appropriate quarantine measures. China is one of the countries in the world suffering the most from the invasion of alien species (Xie et al. 2001). In order to prevent their entry into China, the Ministry of Agriculture and Rural Affairs of the People’s Republic of China has issued a catalog of quarantine pests for plants imported into China (http://dzs.customs.gov.cn/dzs/2746776/3699554/index.html; accessed 12 Dec 2024). This catalogue includes *Alternariatriticina* and *Plenodomuslibanotidis* (Synonym *Leptosphaerialibanotidis*), both of which can be spread over long distances via plant seeds, bulk feed grains, soil, etc., and can even be spread with cargo carried by passengers.

*Alternariatriticina* can cause leaf blight of wheat, especially in central and eastern India, and in severe cases, the damage to wheat yield can reach 46–75% (Raut et al. 1983). In addition, it can also infect a variety of plants such as oats, barley, rye and sesame, seriously threatening crop production (Zhao et al. 2021). This pathogen has been regarded as a quarantine or high-risk pathogen in many countries including Australia, Brazil, China, New Zealand, and South Africa (Perelló and Sisterna 2006; Plant Health Australia 2009; Zhao et al. 2021). *Plenodomuslibanotidis*, known for causing brown rot in *Apiaceae*, has been reported in South Africa, Germany, Russia, Sweden, and Switzerland (Baramidze et al. 2015; Zhao et al. 2021). Of particular concern is its role as a major pathogen affecting carrots in northern Europe. Therefore, it is crucial to prevent its introduction into China, the world’s leading producer of carrots (Zhao and Zhang 2020), and other non-endemic regions. However, as far as we know, no specific, sensitive, rapid, and effective molecular detection assays for these two species have been developed to date.

One of the recent advances in the detection of invasive species is the integration of recombinase polymerase amplification (RPA) with CRISPR-based technologies, offering improved accuracy, speed, sensitivity, and ease over traditional methods (Gootenberg et al. 2018). This method allows for isothermal reactions at 37–42 °C within one hour, making it ideal for portable, rapid nucleic acid detection (Gootenberg et al. 2018). The detection method takes advantage of the collateral nuclease activity of CRISPR-associated proteins, such as Cas12 and Cas13, which recognize and cleave target nucleic acids while simultaneously cleaving ssDNA reporter probes (Gootenberg et al. 2017). Notably, the CRISPR/Cas12a system precisely detects double-stranded DNA with collateral reporter probe cleavage, representing a new generation of rapid and precise nucleic acid detection (Chen et al. 2018). Commonly integrated signal reporter systems include paper-based lateral flow strips (PLFS) and fluorescence-based detection systems (FRB). This platform has shown efficacy in detecting several pathogenic fungi such as *Diaportheaspalathi*, *Diaporthecaulivora*, *Fusariumverticillioides*, *Magnaportheoryzae Triticum* (MoT) pathotype, *Setophomaterrestris*, and *Verticilliumdahliae* (Kang et al. 2021; Wang et al. 2023; Liang et al. 2024; Sun et al. 2024; Zhao et al. 2024).

In this study, we developed a rapid, equipment-independent and on-site visualization assay combining RPA with CRISPR/Cas12a for two high-risk quarantine pathogens, i.e. *A.triticina* and *P.libanotidis*.

## ﻿Materials and methods

### ﻿Sample collection

The DNA of *Alternariatriticina* (CBS 763.84), *Plenodomusconfertus* (CBS 375.64), *P.congestus* (CBS 244.64), *P.enteroleucus* (CBS 142.84), *P.hendersoniae* (CBS 113702), *P.libanotidis* (CBS 113795), *P.lindquistii* (CBS 386.80), *P.lingam* (CBS 275.63, CBS 147.24, CBS 260.94), *P.pimpinellae* (CBS 101637), *P.tracheiphilus* (CBS 127250), *P.tracheiphilus* (CBS 551.93) was obtained from the culture collection (CBS) of the Westerdijk Fungal Biodiversity Institute in the Netherlands and used for molecular diagnosis. Other strains were collected from our previous work (See Suppl. material 1: table S1) and preserved in CGMCC and the LC culture collection (a personal culture collection of Lei Cai, housed in the Institute of Microbiology, Chinese Academy of Sciences).

For validation experiments of the RPA-CRISPR/Cas12a assay on simulated infected plant tissues, we selected the main hosts of the two pathogens, namely wheat and carrot. Healthy wheat samples were collected from fields in Xizang Autonomous Region in China, and carrots were bought from a supermarket in Beijing.

### ﻿DNA extraction

The strains were grown at 25 °C for 7 days, and the hyphae were scraped with a scalpel into the 2 mL centrifuge tube. For genomic DNA extraction of strains and plant tissues, we used a modified CTAB protocol (Phurbu et al. 2024). DNA concentration was quantified using NanoDrop™One (Thermo Fisher Scientific, Waltham, MA, USA).

### ﻿Design of primers and crRNA

To enhance the detection accuracy of the target species (*A.triticina* and *P.libanotidis*), we retrieved a comprehensive set of sequences for *Alternaria* spp., *Plenodomus* spp., and their phylogenetically related species from the NCBI GenBank database. These sequences were subsequently utilized for the design of species-specific primers and crRNA targeting each of the aforementioned species (See Suppl. material 1: table S2). By analyzing the sequences of the target species and their relatives, we clarified that the *gapdh* sequences were used to design specific primers for *A.triticina*, and *tub2* sequences were used for *P.libanotidis*.

The sequences were aligned using MAFFT v. 7 (http://mafft.cbrc.jp/alignment/server/index.html). After that, the conservative region of target species and the variant region for other species were chosen to design primer pairs according to the manual of TwistAmp^®^ DNA Amplification Kits. It is noteworthy that, the target amplicon should contain at least one protospacer adjacent motif (PAM) site (5′-TTTN-3′) to facilitate recognition by Cas12a (Chen et al. 2018).

For Cas12a cleavage, the crRNA used in the CRISPR detection system was designed based on the amplified product derived from the target species (See Suppl. material 1: figs S1, S2) and synthesized by Sangon Biotech. The FAM/BHQ1-labeled single-stranded DNA (ssDNA) was employed and cleaved for the RPA-Cas12a-mediated real-time and end-point fluorescence assay. In contrast, a FAM/Biotin-labeled ssDNA was utilized for lateral flow strip detection. The complete sequence of the crRNA included a direct repeat sequence for Cas12a recognition 5′-UAAUUUCUACUAAGUGUAGAU-3′ (scaffold sequence) and a specific spacer sequence (guide sequence) for each species (See Suppl. material 1: figs S1, S2).

### ﻿RPA assay optimization

The RPA assay was performed using the TwistAmp™ Basic Kit (TwistDx™, Cambridge, United Kingdom). The RPA reaction mixture, with a total volume of 50 μL, was performed by combining 29.5 μL rehydration buffer, 12.2 μL DEPC-H_2_O, 2.4 μL of each forward and reverse primer (10 μmol/L), 1 μL template DNA, and 2.5 μL magnesium acetate (280 mmol/L) in the manufacture-provided reaction tubes containing recombinase, polymerase, and single-strand binding protein. The reaction was carried out at 39 °C for 30 min in a T30D tri-block super-gradient PCR system (LongGene, Hangzhou, Zhejiang, China), followed by assessment of the amplification products via 2% agarose gel electrophoresis.

To establish optimal RPA reaction conditions, a systematic optimization approach was implemented. Initially, all other parameters were held constant, the incubation time was varied (set at 5, 10, 15, 20, 30 min) to determine the optimal reaction time based on band brightness. Subsequently, with the optimized incubation time and other parameters held constant, the RPA temperature was adjusted (set at 33, 35, 37, 39, 41 °C) to determine the optimal temperature based on band brightness (Zhao et al. 2024).

### ﻿CRISPR/Cas12a-FRB detection

The RPA-CRISPR/Cas12a-FRB assay system contains 2 μL of the above RPA product, 14.4 μL DEPC-H_2_O, 2 μL NEBuffer (10×), 0.4 μL Cas12a (5 μmol/L, New England Biolabs, Ipswich, MA, USA), 0.4 μL crRNA (0.01 mmol/L), 0.8 μL FQ-DNA reporter (5 μmol/L). After incubating at 37 °C for 30 min to perform CRISPR/Cas12a cleavage assay, the products were detected directly by the naked eye under blue light. Positive reactions were indicated by visible fluorescent signals, whereas negative reactions were transparent and colorless.

To optimize the reaction conditions of the CRISPR/Cas12a-FRB detection system, the concentration ratio of Cas12a to crRNA was systematically adjusted (0 nM/0 nM, 50 nM/100 nM, 100 nM/200 nM, 150 nM/250 nM, 200 nM/300 nM), with subsequent observation of the fluorescence signal intensity under each condition. Subsequently, the concentration of the fluorescent reporter (FQ-DNA) was optimized through a series of dilutions (50, 100, 200, 400, 800 nmol/L), while maintaining other parameters constant (Zhao et al. 2024). Finally, the reaction time was optimized by varying the incubation time at 5, 10, 15, 20, 30 min, followed by the evaluation of fluorescence signal intensity under different time conditions.

Using the optimized reaction conditions obtained above, the sensitivity of the RPA-CRISPR/Cas12a-FRB assay was further evaluated. DNA was serially diluted 10-fold to concentrations of 1 ng/μL, 0.1 ng/μL, 10 pg/μL, 1 pg/μL, 0.1 pg/μL, and 0.01 pg/μL. Each sample was tested in triplicate with sterile water as the negative control.

### ﻿CRISPR/Cas12a-PLFS detection

The RPA-CRISPR/Cas12a-PLFS assay system comprises 2 μL RPA product, 13.2 μL DEPC-H2O, 2 μL NEBuffer (10×), 0.4 μL Cas12a (5 μmol/L, New England Biolabs, Ipswich, MA, USA), 0.4 μL crRNA (0.01 mmol/L), and 2 μL LF-DNA reporter (5 μmol/L). Following a 30 min incubation at 37 °C to conduct the CRISPR/Cas12a cleavage assay, 80 μL of DEPC-H2O was added into the reaction tube and thoroughly mixed. Subsequently, a lateral flow strip (Suzhou Gendx Biotech Co., Ltd., Suzhou, China) was placed into the tube and left to incubate at room temperature for 7 min. A positive result is indicated by the presence of a red band at the test line or at both the test and control lines on the strip. Conversely, a negative outcome is indicated by the absence of a red band at the test line but the presence of a red band at the control line.

To further optimize the reaction conditions of the CRISPR/Cas12a-PLFS system, the reporter concentration of the 100 μL reaction system was adjusted, with LF-DNA concentrations set at 50, 100, 200, 400, and 800 nmol/L, while keeping other parameters constant. The reaction time was then optimized by varying the incubation period to 5, 10, 15, 20, and 30 min.

Using the optimized reaction conditions obtained above, the sensitivity of the RPA-CRISPR/Cas12a-PLFS assay was further evaluated. DNA was serially diluted 10-fold to concentrations of 1 ng/μL, 0.1 ng/μL, 10 pg/μL, 1 pg/μL, 0.1 pg/μL, and 0.01 pg/μL. Each sample was tested in triplicate with sterile water as the negative control.

### ﻿RPA-CRISPR/Cas12a for detecting pathogens in plant samples

As obtaining field plant samples infected with quarantine fungi is challenging in China, to validate the effectiveness of the detection method in environmental sample testing, this study employed a simulation method by mixing the DNA of quarantine fungi with that of their respective main hosts in specified proportions. Healthy wheat samples were collected from fields in Xizang Autonomous Region, China, while carrots were acquired from a local supermarket in Beijing, China. Subsequently, the DNA was extracted from at least three samples of each plant type. We then combined wheat DNA with *A.triticina* DNA, and carrot DNA with *P.libanotidis* DNA. The final DNA concentrations of the three pathogens were set to 1 ng/μL, 0.1 ng/μL, 10 pg/μL, 1 pg/μL, 0.1 pg/μL, and 0.01 pg/μL, respectively. Three replicates were set up for each simulation sample. The genomic DNA of the two target pathogens was used as the positive control, while sterile water was utilized as the negative control. Finally, the optimized reaction conditions of RPA-CRISPR/Cas12a-FRB and RPA-CRISPR/Cas12a-PLFS determined above were implemented to detect the presence of the pathogens in the simulated plant samples.

## ﻿Results

### ﻿Specificity of RPA and RPA-CRISPR/Cas12a primers and crRNA

**Figure 1.**
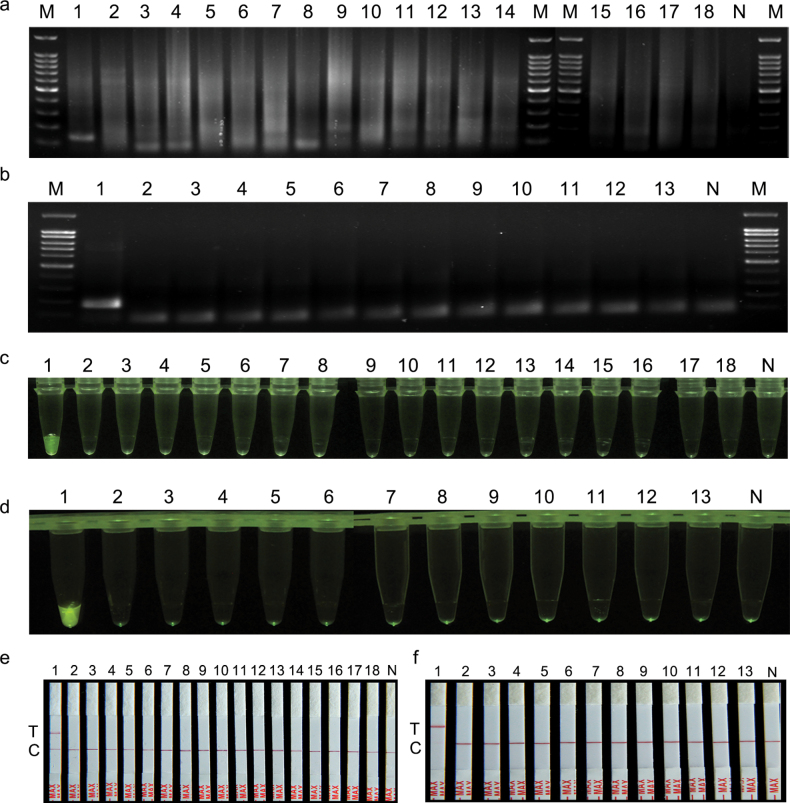
**a–f** specificity of RPA and RPA-CRISPR/Cas12a primers for *A.triticina* (**a, c, e**) and *P.libanotidis* (**b, d, f**). **a, b**RPA detection assay; M: 100 bp DNA ladder; N: ddH_2_O; **c, d**RPA-CRISPR/Cas12a-FRB; **e, f**RPA-CRISPR/Cas12a-PLFS assay, T: test band, C: control band; **a, c, e** 1–18: *A.triticina* (target species), *A.napiformis*, *A.sesami*, *A.cantlous*, *A.infectoria*, *A.malorum*, *A.ochroleuca*, *A.longipes*, *A.tenuissima*, *A.tenuissima*, *A.ethzedia*, *A.tenuissima*, *A.ethzedia*, *A.abundans*, *A.alternata*, *A.arbusti*, *A.solani*, *A.rosae*; **b, d, f** 1–13: *P.libanotidis* (target species), *P.confertus*, *P. congePius*, *P.enteroleucus*, *P.hendersoniae*, *P.lindquipiii*, *P.pimpinellae*, *P.lingam*, *P.lingam*, *P.lingam*, *P.deqinensis*, *P.biglobosus*, *P.tracheiphilus*.

To evaluate the effect of the specific primers that we designed for *A.triticina* and *P.libanotidis* (Table 1), RPA reaction was initially performed using the strains listed in the Suppl. material 1: table S1. Specifically, when the target species was *A.triticina*, the strains used for primer-specific detection were all *Alternaria* strains in the Suppl. material 1: table S1; when the tested species was *P.libanotidis*, the strains used for primer-specific detection were all *Plenodomus* strains. Agarose gel electrophoresis results showed that the RPA products of the three target species displayed obvious target bands, and the total length of amplified products were 127 bp and 172 bp, respectively; while their related fungal species did not show any amplified band (Fig. 1a, b). Subsequently, we selected a PAM site (5′-TTTG-3′) in the amplified sequence of each target species and used its adjacent sequence as crRNA for the three species (See Suppl. material 1: figs S1, S2).

**Table 1.** Primers, guide RNAs and reporters used in this study.

Objective species	Primer Name	Sequence (5′-3′)	Length	Reaction
*Alternariatriticina*	At-RPA-F	5’-CCCACTACGCTGTAAGCATCCCCGCGCGAAC-3’	32	RPA
	At-RPA-R	5’-GCCTGCGTGTGTTAGCCTGCGTCCTGTAGCG-3’	32	
	At-crRNA	5’-UAAUUUCUACUAAGUGUAGAUCGAUGCCACGGAGUAGUUCUAC-3’	44	
	FQ-DNA	5’-(6-FAM) TTATT (BHQ1)-3’	5	CRISPR/Cas12a-FRB
	LF-DNA	5’-(6-FAM) TTTTTTTTTT (Biotin)-3’	10	CRISPR/Cas12a-PLFS
*Plenodomuslibanotidis*	Plib-RPA-F	5’-GTCTCAAGCAGTCTTCTCCTGCTCGTCGAACCA-3	34	RPA
	Plib-RPA-R	5’-GAAGTTGTCGGGGCGGAAGAGCTGACCGAAG-3	32	
	Plib-crRNA	5’-UAAUUUCUACUCUUGUAGAUCACAGGCCUCUGGCAACAAGU-3	42	
	FQ-DNA	5’-(6-FAM) TTATT (BHQ1)-3’	5	CRISPR/Cas12a-FRB
	LF-DNA	5’-(6-FAM) TTTTTTTTTT (Biotin)-3’	10	CRISPR/Cas12a-PLFS

The specificity of primers and crRNA used in the RPA-CRISPR/Cas12a-FRB and RPA-CRISPR/Cas12a-PLFS assays for *A.triticina* and *P.libanotidis* was then evaluated, respectively. The detection results of the RPA-CRISPR/Cas12a-FRB assay demonstrated distinct fluorescent signals in the amplified products of the two species, whereas no such signals were observed in the amplified products of negative and blank controls (Fig. 1c, d). Similarly, the results of the RPA-CRISPR/Cas12a-PLFS assay showed distinct red bands at the test line of the strip in the amplified products of the two species, contrasting with the negative and blank controls where bands were only observed at the control line (Fig. 1e, f).

### ﻿Optimization of RPA reaction time and temperature

**Figure 2.**
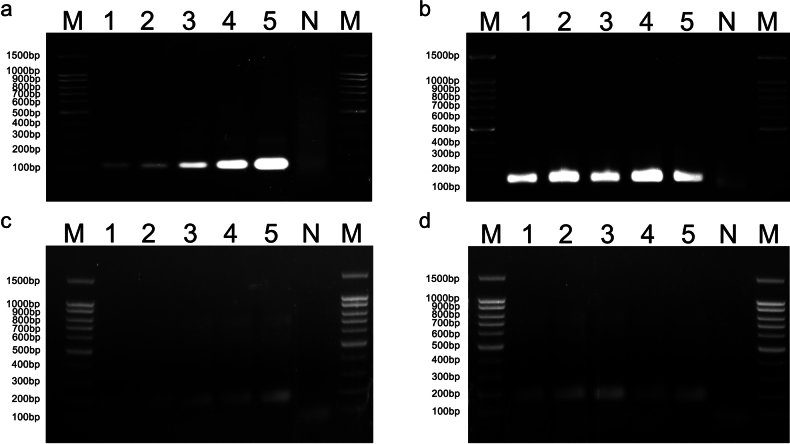
Optimization of RPA reaction time and RPA reaction temperature for *A.triticina* (**a, b**) and *P.libanotidis* (**c, d**); M: 100 bp DNA ladder; N: ddH_2_O; **a, c** optimization of RPA reaction time. 1–5: 5 min, 10 min, 15 min, 20 min, 30 min; **b, d** optimization of RPA reaction temperature. 1–5: 33 °C, 35 °C, 37 °C, 39 °C, 41 °C.

The amplified products from the RPA reactions with different reaction times, 5, 10, 15, 20, and 30 min, were assessed through agarose gel electrophoresis. The results revealed that the brightness of the target bands increased with longer RPA amplification times (Fig. 2a, c), while no distinct bands were observed in the blank controls. While, to meet the requirements of detection efficiency and speed at the same time, 15 min amplification time for *A.triticina* and 20 min for *P.libanotidis* were selected as experimental condition for the subsequent RPA-CRISPR/Cas12a detection system.

Furthermore, the amplified products from RPA reactions conducted at temperatures of 33, 35, 37, 39, and 41 °C were subjected to agarose gel electrophoresis analysis (Fig. 2b, d). The temperature of 35 °C for *A.triticina* and 37 °C for *P.libanotidis* was chosen as the experimental condition for the subsequent RPA-CRISPR/Cas12a detection system to ensure efficient detection.

### ﻿Optimization of CRISPR/Cas12a-FRB assay

**Figure 3.**
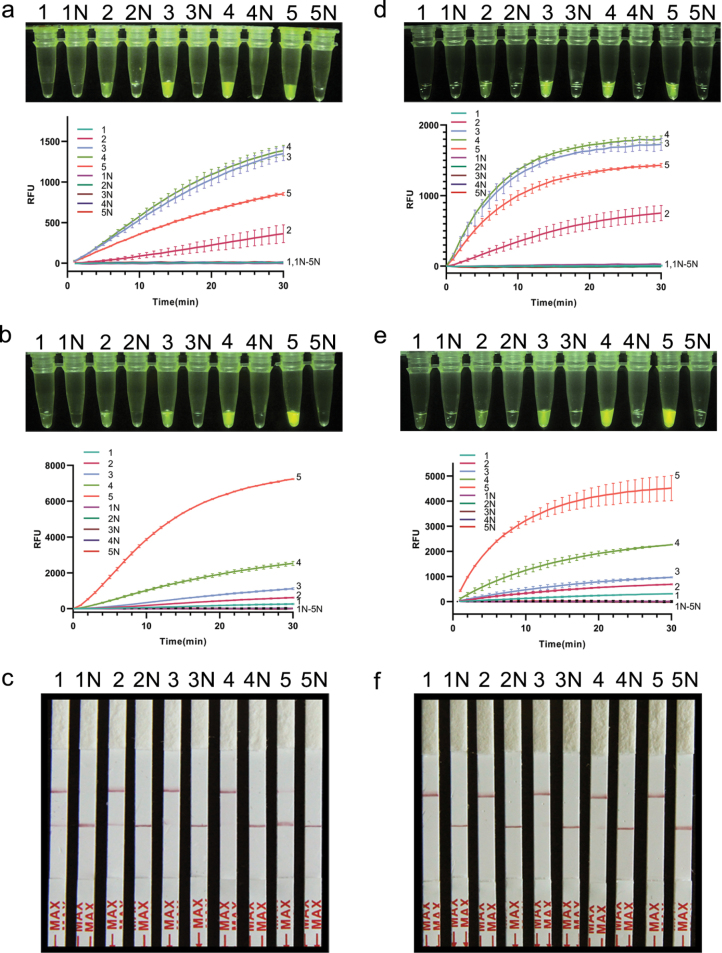
**a, b, d, e**RPA-CRISPR/Cas12a-FRB assay for *A.triticina* (**a**, **b**) and *P.libanotidis* (**d**, **e**). **a, d** optimization of Cas12a/CrRNA concentrations for visual detection and their real-time fluorescent signals. 1–5: The ratio of Cas12a/crRNA, 0 nM/0 nM, 50 nM/100 nM, 100 nM/200 nM, 150 nM/250 nM, 200 nM/300 nM, respectively; 1N–5N: ddH_2_O; **b, e** optimization of FQ-DNA reporter molecule concentrations in the reaction system and their real-time fluorescent signals. 1–5: 50 nmol/L, 100 nmol/L, 200 nmol/L, 400 nmol/L, 800 nmol/L, respectively; 1N–5N: ddH_2_O. **c, f** optimization of LF-DNA reporter molecule concentrations in the RPA-CRISPR/Cas12a-PLFS assays for *A.triticina* (**c**) and *P.libanotidis* (**f**). 1–5: 50 nmol/L, 100 nmol/L, 200 nmol/L, 400 nmol/L, 800 nmol/L, respectively; 1N–5N: ddH_2_O.

In the CRISPR/Cas12a-FRB detection system for *A.triticina* and *P.libanotidis*, the reaction solution appeared colorless and transparent when the Cas12a to crRNA concentration ratio was 0 nM/0 nM, and the fluorescence signal exhibited a generally incremental trend with the rising ratio of Cas12a to crRNA (Fig. 3a, d). However, the fluorescence signal plateaued after reaching a certain threshold concentration ratio. For example, in the detection of *A.triticina*, beyond the ratio of 100 nM/200 nM, there was no discernible increase in fluorescence signal intensity even with further increments in the concentration ratio (Fig. 3a). To optimize experimental costs, we determined the ratio of 100 nM/200 nM as the optimal reaction condition for subsequent experiments related to *A.triticina*. Similarly, for *P.libanotidis*, ratio of 100 nM/200 nM was identified as the optimal choices for subsequent reactions (Fig. 3d).

The optimization study of fluorescent reporter molecule concentrations revealed that at 50 and 100 nmol/L, the FQ-DNA reporter molecule triggered subtle fluorescence signals in the CRISPR/Cas12a-FRB detection system for the two species (Fig. 3b, e). With an increase in the concentration of the reporter molecule, there was a corresponding rise in fluorescence intensity. Striking a balance between cost-effectiveness and brightness, this research settled on a final concentration of 400 nmol/L for the optimal detection of *A.triticina* and *P.libanotidis* (Fig. 3b, e).

**Figure 4.**
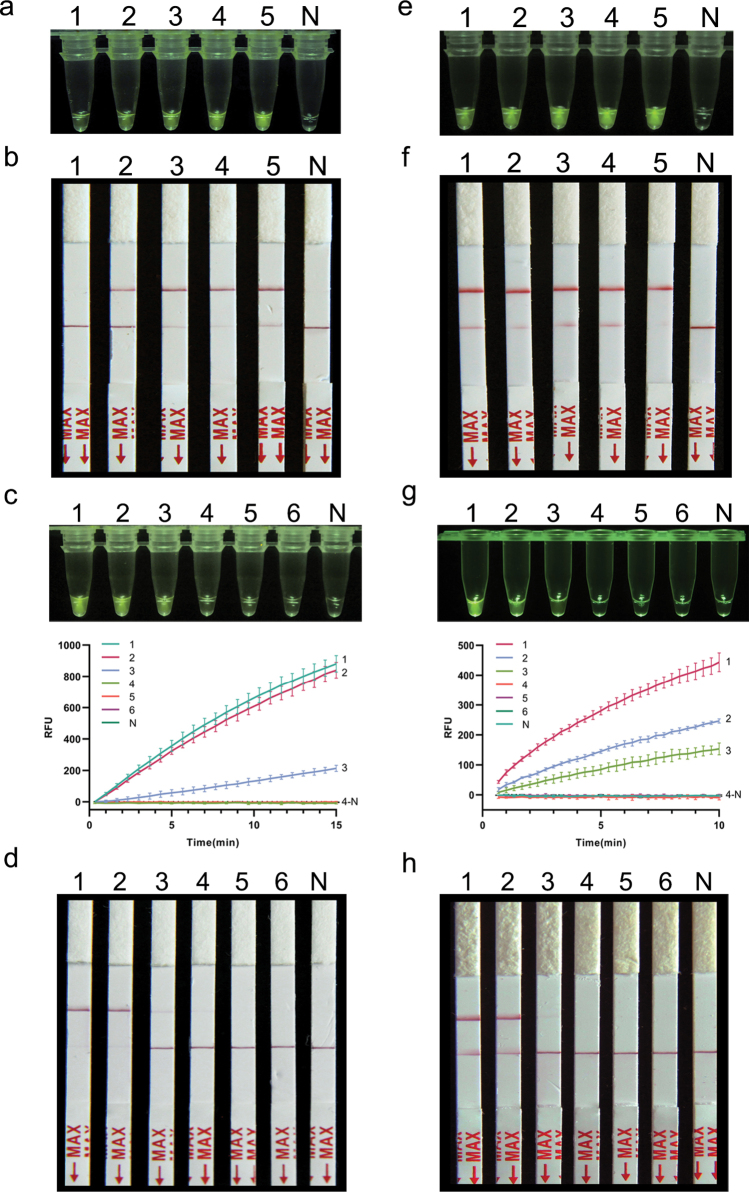
**a, e** optimization of reaction time of visual detection and the real-time fluorescent signals in the RPA-CRISPR/Cas12a-FRB assays for *A.triticina* (**a**) and *P.libanotidis* (**e**). 1–5: 5 min, 10 min, 15 min, 20 min, 30 min, respectively; N: ddH_2_O. **b, f** optimization of reaction time for visual detection in the RPA-CRISPR/Cas12a-PLFS assays for *A.triticina* (**b**) and *P.libanotidis* (**f**). 1–5: 5 min, 10 min, 15 min, 20 min, 30 min, respectively; N: ddH_2_O. **c, g** sensitivity verification of RPA-CRISPR/Cas12a-FRB visual detection system assay for *A.triticina* (**c**) and *P.libanotidis* (**g**). 1–6: 1 ng/μL, 0.1 ng/μL, 10 pg/μL, 1 pg/μL, 0.1 pg/μL, 0.01 pg/μL, respectively. N: ddH_2_O. **d, h** sensitivity verification of RPA-CRISPR/Cas12a-PLFS visual detection system for *A.triticina* (**d**) and *P.libanotidis* (**h**). 1–6: 1 ng/μL, 0.1 ng/μL, 10 pg/μL, 1 pg/μL, 0.1 pg/μL, 0.01 pg/μL, respectively. N: ddH_2_O.

Further, we optimized the reaction time for the CRISPR/Cas12a-FRB system. In the detection system for *A.triticina*, a faint fluorescent signal could be detected when the reaction time was 5 min. Quantitative analysis revealed a time-dependent enhancement of fluorescence intensity, with a statistically significant increase (*p* < 0.05) observed with prolonged reaction durations. Notably, robust fluorescence signals became visibly detectable without instrumentation after 15 min of reaction time (Fig. 4a). The *P.libanotidis* detection system exhibited superior sensitivity, with detectable fluorescence signals emerging within 5 min of reaction initiation, establishing this duration as the standard reaction time for *P.libanotidis* detection (Fig. 4e).

### ﻿Optimization of CRISPR/Cas12a-PLFS assay

Similar to the CRISPR/Cas12a-FRB detection system, the concentration of reporter molecule and reaction time of CRISPR/Cas12a-PLFS were optimized. Upon achieving a terminal concentration of 50 nmol/L for the LF-DNA reporter molecule, red bands are visible on the test strip of *A.triticina* and *P.libanotidis* (Fig. 3c, f). Therefore, to avoid the addition of overly saturated LF-DNA reporter molecules, we ultimately determined 50 nmol/L as the optimal concentration for the detection assay. In addition, the reaction time of 10 min and 5 min was selected to detect *A.triticina* and *P.libanotidis*, respectively (Fig. 4b, f).

### ﻿Sensitivity of RPA-CRISPR/Cas12a assay

To evaluate the sensitivity of RPA and RPA-CRISPR/Cas12a systems, we used different concentrations of genomic DNA. The results show that 1 ng/μL DNA is the detection limit of the RPA system for the two species (not shown), and the addition of the CRISPR/Cas12a detection system notably improves the sensitivity of DNA detection. Both RPA-CRISPR/Cas12a-FRB and RPA-CRISPR/Cas12a-PLFS showed detectable signals at a DNA concentration of 10 pg/μL for the two species (Fig. 4c, d, g, h). As the DNA concentration was further reduced, the fluorescent signal and the red band on the test strip became undetectable.

### ﻿Application of RPA-CRISPR/Cas12a assay in simulated plant samples

**Figure 5.**
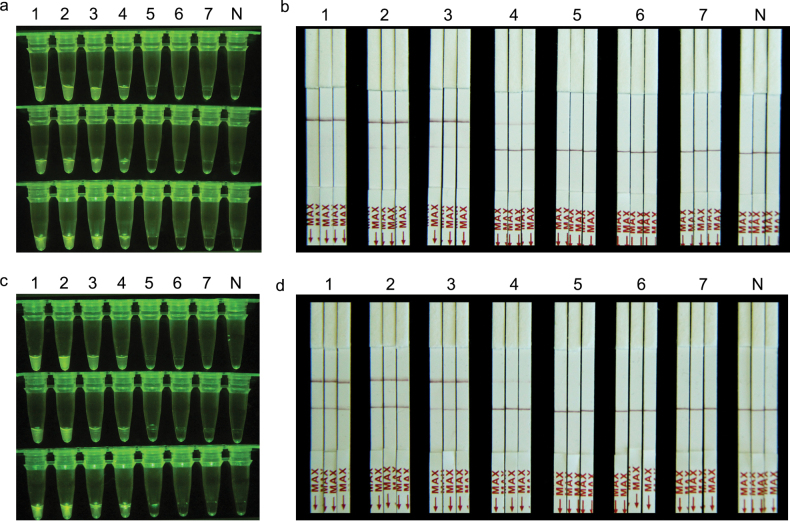
Detection of *A.triticina* (**a, b**) and *P.libanotidis* (**c, d**) in plant samples using RPA-CRISPR/Cas12a assay. **a, b** 1: *A.triticina*; 2–6: The DNA mixture of *A.triticina* and healthy wheat includes DNA concentrations of *A.triticina* at 1 ng, 0.1 ng, 10 pg, 1 pg, 0.1 pg, respectively; 7: Healthy wheat DNA; N: ddH_2_O; **c, d** 1: *P.libanotidis*; 2–6: The DNA mixture of *P.libanotidis* and healthy carrot includes DNA concentrations of *P.libanotidis* at 1 ng, 0.1 ng, 10 pg, 1 pg, 0.1 pg, respectively; 7: Healthy carrot DNA; N: ddH_2_O.

The feasibility of the developed RPA-CRISPR/Cas12a assays for plant samples was evaluated using DNA mixture of carrot, wheat and their respective pathogens. The results showed that the DNA sensitivity of both RPA-CRISPR/Cas12a-FRB and RPA-CRISPR/Cas12a-PLFS assays for the two fungal species is 10 pg/μL, while no such signal was observed in the amplified products when the pathogens’ DNA concentration was further reduced (Fig. 5).

## ﻿Discussion

The spread of plant pathogens in the context of the trade globalization poses a major threat to food and ecological security (Li et al. 2023). At customs ports, the conventional detection process typically involves the isolation and culturing of pathogens, followed by barcode amplification and sequencing, or species identification using real-time PCR. However, sequence analysis and real-time PCR require skilled operators and expensive equipment and are time-consuming and challenging to interpret the results of the analysis (Kralik and Ricchi 2017). Moreover, methods reliant on sequencing and sequence analysis are characterized by long detection times, thereby impeding the efficiency of rapid customs clearance. To mitigate these challenges, scientists have been investigating novel nucleic acid isothermal amplification technologies that significantly reduce equipment requirements. Notably, RPA is one of the fastest developing method, experiencing rapid uptake and market (Li and von Stetten 2019). RPA coupled with CRISPR/Cas12a system has gradually been applied in pathogen detection, offering advantages such as accurate results, high sensitivity, low false positives, simplicity in operation, and visualized results (de Dieu Habimana et al. 2022; Mao et al. 2023; Wani et al. 2024). In this study, detection methods for two important pathogenic fungi were developed using this technology, allowing accurate detection of *A.triticina* and *P.libanotidis* within 30 minutes (Table 2).

**Table 2.** Optimized reaction system for RPA-CRIPSR/Cas12a in this study.

Optimized reaction system		*A.triticina*	Simulated plant tissue	*P.libanotidis*	Simulated plant tissue
RPA	Temperature	35 °C	/	37 °C	/
	Reaction time	15 min	/	20 min	/
	Sensitivity	1 ng	/	1 ng	/
CRISPR/Cas12a-FRB	Cas12a/crRNA	100/200 nmol/L	/	100/200 nmol/L	/
	FQ-DNA	400 nmol/L	/	400 nmol/L	/
	Temperature	37 °C	/	37 °C	/
	Reaction time	15 min	/	5 min	/
	Sensitivity	10 pg	10 pg	10 pg	10 pg
CRISPR/Cas12a-PLFS	Cas12a/crRNA	100/200 nmol/L	/	100/200 nmol/L	/
	LF-DNA	50 nmol/L	/	50 nmol/L	/
	Temperature	37 °C	/	37 °C	/
	Reaction time	10 min	/	5 min	/
	Sensitivity	10 pg	10 pg	10 pg	10 pg
RPA-CRISPR/Cas12a-FRB	Reaction time	30 min	30 min	25 min	25 min
RPA-CRISPR/Cas12a-PLFS	Reaction time	25 min	25 min	25 min	25 min

Specific sequences are fundamental to the development of accurate species identification techniques, with precise species information being a crucial prerequisite for defining specific sequences. Although specific primer LJY1/LJY2 was designed for *A.triticina* based on ITS (Zhang et al. 2016), its reliability requires further validation. This is because the phylogenetic position of *A.triticina* was ambiguous when designing the specific primer, and the ITS sequences of *A.triticina* generated from type strains stored in different culture collections are inconsistent, namely the ITS sequence of MUCL 44210 / IMI 289962 / E.G.S. 17-061 (AY714476) (Mercado Vergnes et al. 2006) differs by 9 nucleotides from AY762948 (E.G.S. 17-061) (Xue and Zhang 2007). Consequently, ensuring the reliability of detection techniques initially requires clarifying and verifying the accuracy of the target sequences utilized for primer design. Until recently, Zhao et al. (2021) clarified the systematic position of *A.triticina* using type strain, which lay an important research foundation for the development of rapid detection techniques. Nevertheless, this study revealed that the ITS sequences of *A.triticina* and its close relatives are highly similar, making it impossible to design species-specific primers. Hence, primers specific of *A.triticina* were designed using the *gapdh* gene in this study, which exhibits obvious interspecies differences.

We acknowledge the critical need to consider intraspecific variation in primer design. However, only a single *tub2* sequence (KY064059) for *P.libanotidis* is publicly available (NCBI), corresponding to strain CBS 113795. This strain, used in our study, is also the only strain of *P.libanotidis* preserved in many fungal culture collections such as CBS, CGMCC, and LC. Given the importance and urgency of establishing detection methods for this pathogen, we weighed the pros and cons and decided to design species-specific primers based on this single available strain. We aim to validate its specific primer further once additional strains become accessible through future international collaborations.

In the RPA-CRISPR/Cas12a assay, increasing the number of copies of the target DNA through extending the RPA reaction time can enhance the fluorescence signal of the CRISPR/Cas12a detection system, thereby improving the detection sensitivity (Lillis et al. 2016). However, simultaneously, it will extend the overall RPA-CRISPR/Cas12a detection process. Therefore, to simultaneously balance detection effectiveness and duration, the RPA reaction time was evaluated based on the gel electrophoresis band brightness of the RPA reaction product (Zhao et al. 2024). Consequently, a 15-min RPA reaction time was chosen for *A.triticina*, and a 20-min time for *P.libanotidis* in the subsequent CRISPR/Cas12a detection system (Table 2). In addition, RPA reaction time and the concentration of the fluorescent reporter will affect the sensitivity, signal intensity and detection time of the RPA-CRISPR/Cas12a assay (Zhao et al. 2024). However, in order to consider both time and economic costs, we cannot increase the reaction time and reagent concentration indefinitely. If there is a higher detection requirement for sensitivity, the RPA reaction time and reporter molecule concentration can be appropriately increased.

To date, the PRA-CRISPR/Cas12a detection system has shown significant promise in diagnostics. Although the per-reaction cost of the RPA-CRISPR/Cas12a-FRB or RPA-CRISPR/Cas12a-PLFS is higher than that of qPCR (Zhao et al. 2024), this system requires less expensive or even no equipment, which helps offset some of the additional costs. We acknowledge certain limitations in this study. Firstly, due to challenges in obtaining materials of quarantine fungi, the number of samples used to test primer specificity was limited. However, in designing the primers, we downloaded as many sequences as possible for quarantine species and their closely related counterparts from public databases. Sequence alignment analyses showed that the primers are both highly specific between species and conserved within species, providing strong evidence for their specificity and effectiveness. Therefore, we are confident in the reliability of the primers and plan to further validate the RPA-CRISPR/Cas12a detection method through international collaboration.

Secondly, since the two quarantine fungi are not present in China, it was difficult to obtain field plant samples. However, by simulating field conditions through the mixing of plant tissue and pathogen DNA, we were able to validate the feasibility of the detection method for identifying plant samples infected with pathogenic fungi. In the future, we will actively seek to establish collaborations with international research institutions or official quarantine agencies that have access to relevant resources. This will enable us to utilize naturally infected field samples or actual quarantine-intercepted samples containing the target quarantine pathogenic fungi to rigorously evaluate the effectiveness of the detection method developed in this study. Concurrently, a key focus will be the development or adaptation of simplified DNA extraction methods suitable for rapid on-site detection, in order to fully leverage the speed advantage of the RPA-CRISPR assay.

Thirdly, it is important to note that the RPA-CRISPR/Cas12a visual detection system developed in this study is designed for qualitative detection, rather than quantitative detection. Lastly, to our knowledge, while current researches on detection methods for plant pathogenic fungi typically involves optimizing reaction factors—such as temperature, time, and probe concentrations—one at a time (or sequentially), this approach does not account for potential interactions between these factors. Future work could benefit from introducing more systematic experimental design approaches to achieve more efficient and comprehensive optimization.

## ﻿Additional information

### Adherence to national and international regulations

All the fungal strains used in this study have been legally obtained, respecting the Convention on Biological Diversity (Rio Convention).

### Author ORCIDs

Lei Cai https://orcid.org/0000-0002-8131-7274

Fang Liu https://orcid.org/0000-0002-8489-5926

### Data availability

All of the data that support the findings of this study are available in the main text or Supplementary Information.

## Supplementary materials

Supplementary material 1 Additional information Data type docx Explanation note **fig. S1.** Alignment of *gapdh* sequences of *Alternaria* species. **a.** Location of RPA primers (blue arrows), PAM region (red), crRNA binding sites (yellow) of *A.triticina*. The direction of the blue arrows indicates the direction of RPA primers. **b.** A detailed schematic showing the binding of crRNA from *A.triticina* to the target gene fragments. **fig. S2.** Alignment of *tub2* sequences of *Plenodomus* species. **a.** Location of RPA primers (blue arrows), PAM region (red), crRNA binding sites (yellow) of *P.libanotidis*. The direction of the blue arrows indicates the direction of RPA primers. **b.** A detailed schematic showing the binding of crRNA from *P.libanotidis* to the target gene fragments. **table S1.** Fungal strains used for molecular detection in this study. **table S2.** Fungal strains used for primer designation in this study. https://binary.pensoft.net/file/1349565This dataset is made available under the Open Database License (http://opendatacommons.org/licenses/odbl/1.0/). The Open Database License (ODbL) is a license agreement intended to allow users to freely share, modify, and use this Dataset while maintaining this same freedom for others, provided that the original source and author(s) are credited.Dorji Phurbu, Zhipeng Feng, Lei Cai, Fang Liu
