# Supplementary material for: ﻿Rapid and sensitive diagnosis of plant quarantine fungi Alternariatriticina and Plenodomuslibanotidis based on the RPA-CRISPR/Cas12a system
Source: IMA Fungus. 2025 Jun 10;16:e153604. doi: 10.3897/imafungus.16.153604 (PMC12177518; doi:10.3897/imafungus.16.153604)
Supplement: Supplementary material 1 — Additional information [file imafungus-16-e153604-s001.docx]

**Supplementary files**


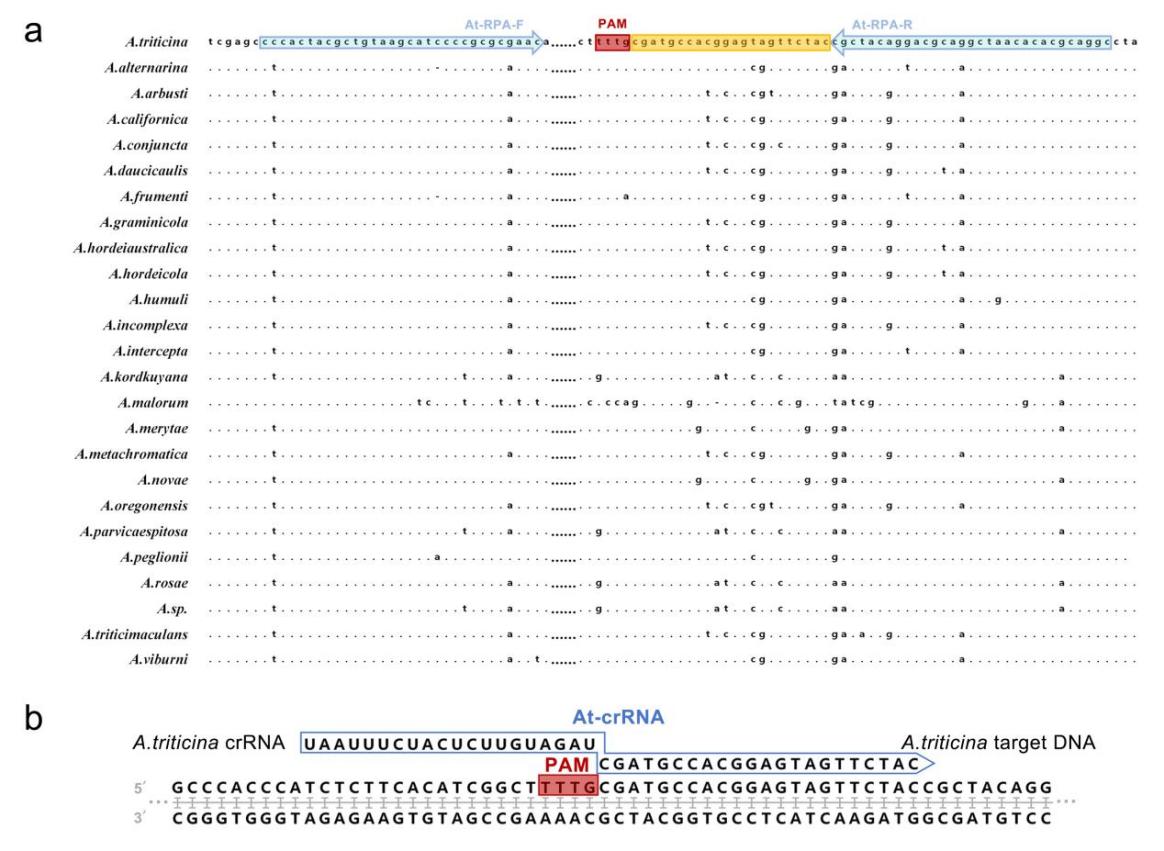


**Fig. S1** Alignment of *gapdh* sequences of *Alternaria* species. **a.** Location of RPA primers (blue arrows), PAM region (red), crRNA binding sites (yellow) of *A. triticina*. The direction of the blue arrows indicates the direction of RPA primers. **b.** A detailed schematic showing the binding of crRNA from *A. triticina* to the target gene fragments.


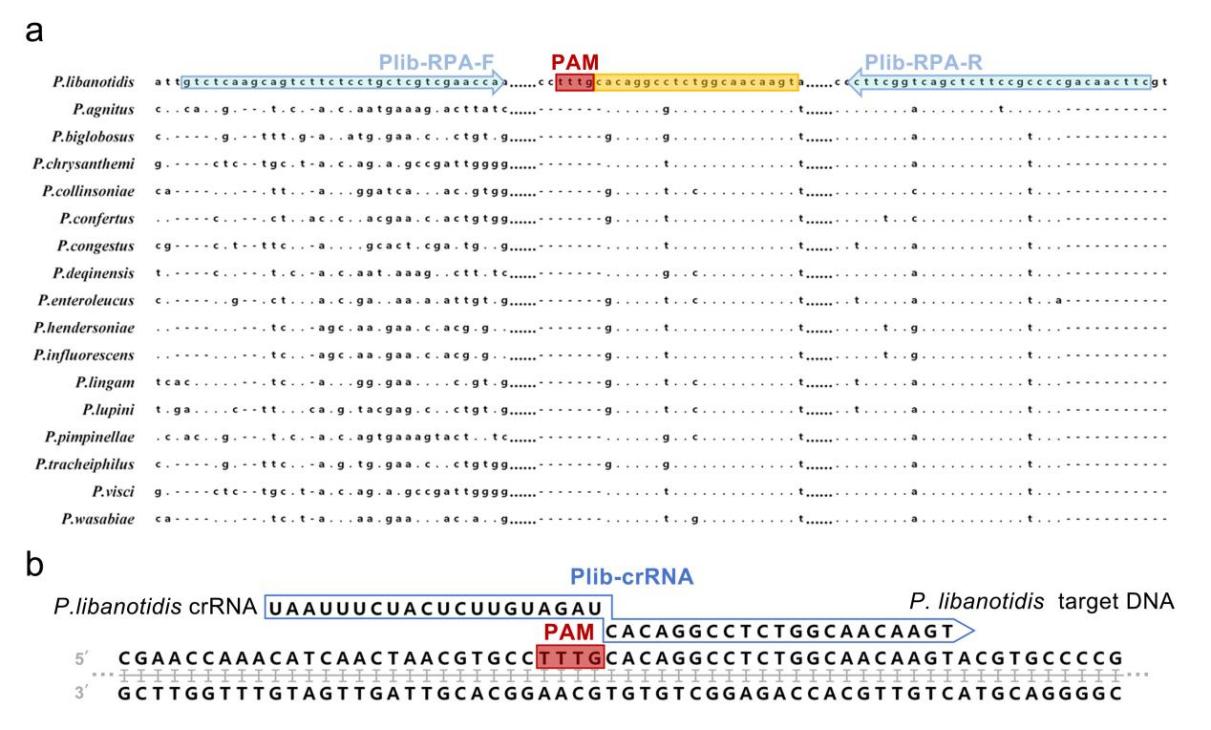


**Fig. S2** Alignment of *tub2* sequences of *Plenodomus* species. **a.** Location of RPA primers (blue arrows), PAM region (red), crRNA binding sites (yellow) of *P. libanotidis*. The direction of the blue arrows indicates the direction of RPA primers. **b.** A detailed schematic showing the binding of crRNA from *P. libanotidis* to the target gene fragments.

**Table S1.** Fungal strains used for molecular detection in this study.

| **Genus** | **Fungal Name** | **Strain Number**1 | **Type** | **Substrate** | **Location** | **Reference** |
| --- | --- | --- | --- | --- | --- | --- |
| ***Alternaria*** | *Alternaria abundans* | LC14953 |  | Triticum sp. | China | This study |
|  | *Alternaria alternata* | LC14933 |  | Triticum sp. | China | This study |
|  | *Alternaria arbusti* | LC14942 |  | Triticum sp. | China | This study |
|  | *Alternaria cantlous* | LC11434 |  | *Poa annua* | America | This study |
|  | *Alternaria ethzedia* | LC14735 |  | Triticum sp. | Kazakhstan | This study |
|  | *Alternaria ethzedia* | LC14888 |  | Triticum sp. | Kazakhstan | This study |
|  | *Alternaria infectoria* | LC11446 |  | *Poa annua* | America | This study |
|  | *Alternaria longipes* | LC14152 |  | *Musa nana* | China | This study |
|  | *Alternaria malorum* | LC11435 |  | *Poa annua* | America | This study |
|  | *Alternaria napiformis* | LC7588 |  | *Unknown* | China | This study |
|  | *Alternaria ochroleuca* | LC11549 |  | *Vitis vinifera* | China | This study |
|  | *Alternaria rosae* | LC18887 |  | *Vicia sepium* | China | This study |
|  | *Alternaria sesami* | LC8306 |  | *Ipomoea aquatica* | China | This study |
|  | *Alternaria solani* | LC15020 |  | *Miscanthus sinensis* | China | This study |
|  | *Alternaria tenuissima* | LC14693 |  | Triticum sp. | China | This study |
|  | *Alternaria tenuissima* | LC14701 |  | Triticum sp. | Canada | This study |
|  | *Alternaria tenuissima* | LC14718 |  | Triticum sp. | Kazakhstan | This study |
|  | *Alternaria triticina* | CBS 763.84 | type | *Triticum aestivum* |  | Zhao et al. 2021 |
| ***Plenodomus*** | *Plenodomus biglobosus* | LC11456 |  | *Brassica campestris* | Canada | This study |
|  | *Plenodomus confertus* | CBS 375.64 |  | *Anacyclus radiatu* | Spain | Zhao et al. 2021 |
|  | *Plenodomus congestus* | CBS 244.64 | type | *Erigeron canadensis* | Spain | Zhao et al. 2021 |
|  | *Plenodomus deqinensis* | CGMCC 3.18221 | type | soil | China | Marin-Felix et al. 2017 |
|  | *Plenodomus enteroleucus* | CBS 142.84 | type | *Catalpa bignonioides* | Netherlands | Zhao et al. 2021 |
|  | *Plenodomus hendersoniae* | CBS 113702 |  | *Salix cinerea* | Sweden | Zhao et al. 2021 |
|  | *Plenodomus libanotidis* | CBS 113795 |  | *Seseli libanotis* | Sweden | Zhao et al. 2021 |
|  | *Plenodomus lindquistii* | CBS 386.80 |  | *Helianthus annuus* | Yugoslavia | Zhao et al. 2021 |
|  | *Plenodomus lingam* | CBS 275.63 |  | Brassica sp. | United Kingdom | Zhao et al. 2021 |
|  | *Plenodomus lingam* | CBS 147.24 |  | Unknown | Unknown | Zhao et al. 2021 |
|  | *Plenodomus lingam* | CBS 260.94 |  | *Brassica oleracea* | Netherlands | Zhao et al. 2021 |
|  | *Plenodomus pimpinellae* | CBS 101637 | type | *Pimpinella anisum* | Israel | Zhao et al. 2021 |
|  | *Plenodomus tracheiphilus* | CBS 127250 |  | *Citrus aurantium* | Italy | Zhao et al. 2021 |
|  | *Plenodomus tracheiphilus* | CBS 551.93 |  | *Citrus limonium* | Israel | Zhao et al. 2021 |

Notes: ^1^CBS: the culture collection (CBS) of the Westerdijk Fungal Biodiversity Institute in the Netherlands; CGMCC: China General Microbiological Culture Collection Center; LC: a personal culture collection of Lei Cai, housed in the Institute of Microbiology, Chinese Academy of Sciences

**Table S2** Fungal strains used for primer designation in this study.

| **Genus** | **Fungal Name** | **Strain Number** | tub21 | gapdh2 |
| --- | --- | --- | --- | --- |
| ***Alternaria*** | *Alternaria alternarina* | EGS 10-193 | / | JQ646289 |
|  | *Alternaria arbusti* | EGS 91-129 | / | JQ693621 |
|  | *Alternaria arbusti* | EGS 91-136 | / | JQ646365 |
|  | *Alternaria californica* | EGS 52-082 | / | JQ646285 |
|  | *Alternaria conjuncta* | BMP 40 | / | AY562401 |
|  | *Alternaria daucicaulis* | EGS 36-1947 | / | JQ646294 |
|  | *Alternaria dianthicola* | CBS 915.96 | / | JQ646282 |
|  | *Alternaria frumenti* | EGS 44-001 | / | JQ646295 |
|  | *Alternaria graminicola* | EGS 41-139 | / | JQ646291 |
|  | *Alternaria hordeiaustralica* | EGS 44-200 | / | JQ646283 |
|  | *Alternaria hordeicola* | EGS 50-184 | / | JQ646284 |
|  | *Alternaria humuli* | EGS 47-140 | / | JQ646293 |
|  | *Alternaria incomplexa* | EGS 17-103 | / | JQ646287 |
|  | *Alternaria intercepta* | EGS 49-137 | / | JQ646297 |
|  | *Alternaria kordkuyana* | KQ-20 | / | MF033826 |
|  | *Alternaria malorum* | CBS 135.31 | / | JQ646278 |
|  | *Alternaria merytae* | EGS 46-153 | / | JQ646292 |
|  | *Alternaria metachromatica* | BMP 45 | / | AY562404 |
|  | *Alternaria novae-zelandiae* | EGS 48-092 | / | JQ646296 |
|  | *Alternaria oregonensis* | EGS 21-194 | / | FJ266491 |
|  | *Alternaria parvicaespitosa* | X1272 | / | MF033842 |
|  | *Alternaria peglionii* | CBS 103.26 | / | JQ646286 |
|  | *Alternaria rosae* | EGS 41-130 | / | JQ646279 |
|  | *Alternaria* sp. | 56492-12FD | / | JQ693635 |
|  | *Alternaria triticimaculans* | EGS 41-050 | / | JQ646280 |
|  | *Alternaria triticina* | CBS 763.84 | / | MW810277 |
|  | *Alternaria triticina* | ATCC 36205 | / | MW945411 |
|  | *Alternaria triticina* | CGMCC 3.9868 | / | MW945413 |
|  | *Alternaria triticina* | CBS 121461 | / | MW945412 |
|  | *Alternaria ventricosa* | EGS 52-075 | / | JQ646290 |
|  | *Alternaria viburni* | EGS 49-147 | / | JQ646288 |
|  | *Embellisia abundans* | CBS 534.83 | / | FJ214852 |
|  | *Embellisia didymospora* | CBS 766.79 | / | JN383470 |
|  | *Lewia ethzedia* | EGS 37-143 | / | AY278795 |
|  | *Lewia infectoria* | EGS 27-193 | / | AY278793 |
| ***Plenodomus*** | *Plenodomus agnitus* | CBS 121.89 | KY064053 | / |
|  | *Plenodomus biglobosus* | CBS 119951 | KY064054 | / |
|  | *Plenodomus chrysanthemi* | CBS 539.63 | KY064055 | / |
|  | *Plenodomus collinsoniae* | CBS 120227 | KY064056 | / |
|  | *Plenodomus confertus* | CBS 375.64 | KY064057 | / |
|  | *Plenodomus congestus* | CBS 244.64 | KY064058 | / |
|  | *Plenodomus deqinensis* | CGMCC 3.18221 | KY064052 | / |
|  | *Plenodomus enteroleucus* | CBS 142.84 | KT266266 | / |
|  | *Plenodomus hendersoniae* | CBS 113702 | KT266271 | / |
|  | *Plenodomus influorescens* | CBS 143.84 | KT266267 | / |
|  | *Plenodomus libanotidis* | CBS 113795 | KY064059 | / |
|  | *Plenodomus lingam* | CBS 260.94 | KY064060 | / |
|  | *Plenodomus lupini* | CBS 248.92 | KY064061 | / |
|  | *Plenodomus pimpinellae* | CBS 101637 | KY064062 | / |
|  | *Plenodomus tracheiphilus* | CBS 551.93 | KT266269 | / |
|  | *Plenodomus visci* | CBS 122783 | KY064063 | / |
|  | *Plenodomus wasabiae* | CBS 120119 | KT266272 | / |

Notes: ^1^*tub2* sequences were used for specific primer designation of *Plenodomus libanotidis*; *^2^gapdh* sequences were used for specific primer designation of *Alternaria tritici*.
